# Cell Model Passports—a hub for clinical, genetic and functional datasets of preclinical cancer models

**DOI:** 10.1093/nar/gky872

**Published:** 2018-09-27

**Authors:** Dieudonne van der Meer, Syd Barthorpe, Wanjuan Yang, Howard Lightfoot, Caitlin Hall, James Gilbert, Hayley E Francies, Mathew J Garnett

**Affiliations:** Wellcome Sanger Institute, Wellcome Genome Campus, Cambridge, CB10 1SA, UK

## Abstract

*In vitro* cancer cell cultures are facile experimental models used widely for research and drug development. Many cancer cell lines are available and efforts are ongoing to derive new models representing the histopathological and molecular diversity of tumours. Cell models have been generated by multiple laboratories over decades and consequently their annotation is incomplete and inconsistent. Furthermore, the relationships between many patient-matched and derivative cell lines have been lost, and accessing information and datasets is time-consuming and difficult. Here, we describe the Cell Model Passports database; cellmodelpassports.sanger.ac.uk, which provides details of cell model relationships, patient and clinical information, as well as access to associated genetic and functional datasets. The Passports database currently contains curated details and standardized annotation for >1200 cell models, including cancer organoid cultures. The Passports will be updated with newly derived cell models and datasets as they are generated. Users can navigate the database via tissue, cancer-type, genetic feature and data availability to select a model most suitable for specific applications. A flexible REST-API provides programmatic data access and exploration. The Cell Model Passports are a valuable tool enabling access to high-dimensional genomic and phenotypic cancer cell model datasets empowering diverse research applications.

## INTRODUCTION

The currently available set of ∼1000 human cancer cell lines are widely used in research and drug development due to their ease of use, broad utility and low reagent costs. Cancer cell lines have made important contributions to the development of anti-cancer drugs, such as the epidermal growth factor receptor (EGFR) inhibitor gefitinib for the treatment of non-small-cell lung cancer (1), inhibitors of the protein kinase BRAF in melanoma (2) and anaplastic lymphoma kinase (ALK) inhibitors for ALK-fusion positive lung cancer (3). They have also been instrumental in research beyond cancer including the development of the polio vaccine (4). Their experimental tractability has led to their use in large-scale genetic and pharmacological screens to identify new drug targets and guide biomarker development including the Genomics of Drug Sensitivity in Cancer (GDSC) project based at the Sanger Institute, the Cancer Cell Line Encyclopedia (CCLE), the National Cancer Institute-60 (NCI-60) cancer cell line screen and the Cancer Therapeutic Response Portal (CTRP) (5–8).

Due to their extensive use over decades, there are several challenges when working with and selecting cancer cell models. Many models have inadvertently been cross-contaminated (9–11) or are associated with numerous synonymous identifiers (11, 12). In addition, key patient and clinical information has been lost including relationships between cell lines originally derived from the same patient or sample (13). The lack of a consistent controlled vocabulary to describe cell line metadata and the large number of synonymous identifiers makes data integration and cross-referencing of datasets burdensome (14, 15). The length of time in culture, culture conditions and exogenous selective pressures on a cell line (e.g. PDX engraftment) can lead to genetic drift (16, 17). Therefore, a clear understanding of the characteristics and source of the model used to generate a given dataset is important for reproducibility of results. Although many cell lines have been genetically and functionally characterized, it is often difficult to determine what information is available for a particular cell line and how to access these data. Furthermore, these datasets are often inaccessible to non-computational, wet-lab scientists. As a result of these issues, the informed selection of cancer models based on patient, clinical and molecular features, and the availability of associated datasets is currently time consuming and difficult.

To address these problems, we have created Cell Model Passports; cellmodelpassports.sanger.ac.uk. The Cell Model Passports is a central portal providing manual or programmatic access to a cancer cell model database containing curated patient, sample and model relationship information as well as genomic and functional datasets. Here, we describe the Passports and available datasets, and provide information on how they can be accessed through a user-friendly web application and programmatically through a REST Application Programming Interface (API).

## MODEL ANNOTATIONS

More than 1200 established cancer cell lines and newly derived organoid models comprise the foundational model set of the Passports, including all those utilized in the Sanger’s GDSC project (5, 18) as well as cell models generated as part of the Human Cancer Models Initiative. The majority of cell models are available to the research community through public repositories. The annotation conducted provides users with key model characteristics and relationships, a summary of relevant genetic features as well as the ability to integrate datasets from multiple resources. Over 30 attributes may be annotated to a model including primary name, synonyms, tissue of origin, disease details including treatment as well as patient information, such as gender, ethnicity and age (Supplementary Table S1).

Cell model names are often the only means to integrate and compare datasets but they are also one of the most variable properties. The Passport model name is accompanied by synonyms and most importantly unique identifiers utilized by established resources such as Research Resource Identifiers (RRID) (19), the Catalogue of Somatic Mutations in Cancer (COSMIC) (20) and CCLE (6) to aid data integration and alignment.

Model lineage, a requirement for tissue-specific analyses, is provided through three progressively granular fields: ‘Tissue’ (*n* = 29 categories), ‘Cancer Type’ (*n* = 44) and ‘Cancer Type Detail’ (*n* = 194). The cancer type descriptors use the NCI-Thesaurus terms, benefitting from established definitions that assist integration with other ontologies. Models in the Passports have been derived from primary, metastatic, pre-cancerous and normal samples as indicated by ‘Tissue Status’, the anatomical site from which the sample was obtained ‘Sample Site’ is also provided.

Annotation of genomic features has been provided for each model via the exploitation of the available genomic datasets. Features include microsatellite stability status, ploidy, mutational burden and the mutation status of 455 cancer driver genes (18). Furthermore, top drug sensitivities for cell models are also provided as determined by the drug profiling of ∼250 compounds as part of the GDSC project (18).

### Model relationships

The annotation of relationships that exist between models is an important feature of the Passports, designed to address the issues faced by researchers of related, mis-identified or cross-contaminated models.

**Figure 1.**
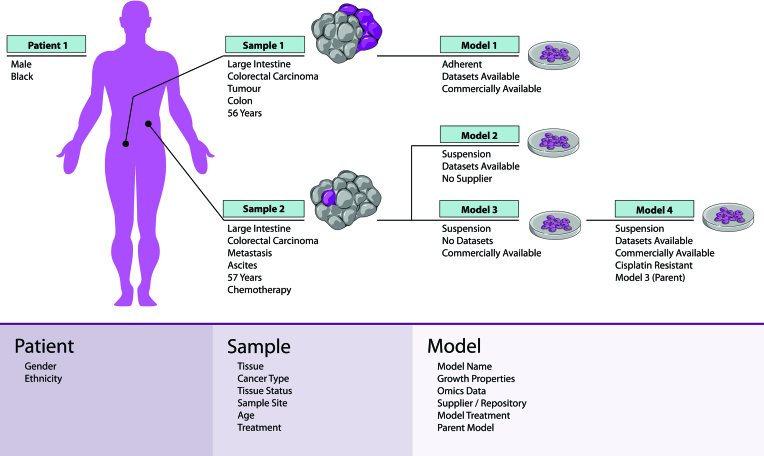
A schematic of the hierarchy used by the Cell Model Passports to capture the relationships that exist between cell models. The patient is at the pinnacle of the hierarchy (Patient 1) followed by the samples obtained (Sample 1 and 2) and models established (Model 1, 2 and 3). Establishment of further cell models from a pre-existing cell model are recorded by a parent-child link (Model 4). Image elements were obtained and adapted from Servier Medical Art under a Creative Commons Attribution 3.0 Unported License.

As outlined in Figure 1, the Passport’s relationship hierarchy captures the key points in the model derivation process, namely the patient, samples obtained and models established. This approach provides users with a clear structure of cell model relationships and the potential to integrate data at any level of the relationship hierarchy. In the database, the hierarchy has been modelled as one-to-many relationships, where a patient can have multiple samples, which can each have multiple models. Any hierarchy between models (e.g. subclones, derived models and drug resistant clones) has been modelled as an adjacency list storing each model’s parent identifier where appropriate.

### Datasets

**Figure 2.**
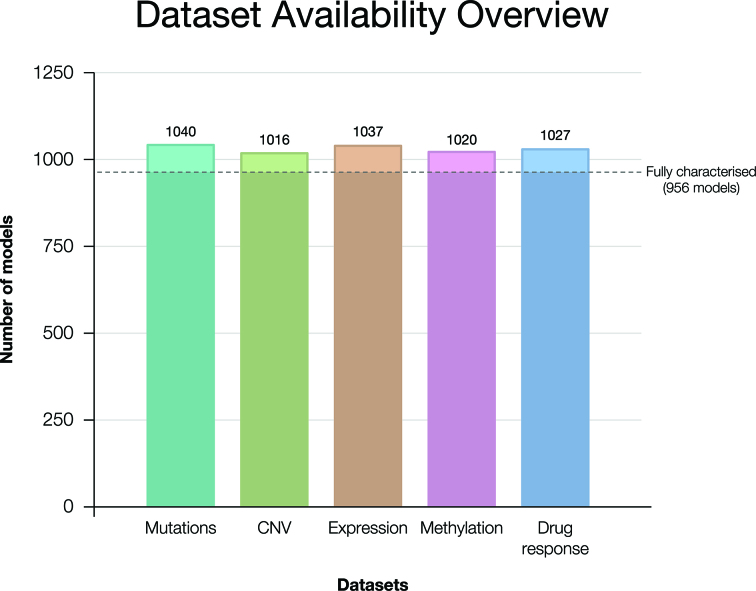
An overview of the availability of various genomic and functional datasets. Currently, mutation, copy number variation (CNV), methylation, gene expression and drug sensitivity datasets are available. To emphasize the lack of sparsity, a subset of 956 models has been highlighted for which all five datasets are available.

Over 3500 genetic datasets and more than 1000 drug sensitivity datasets are available. Most cell models (85%) have associated somatic nucleotide variant, gene expression, copy number variation or methylation datasets (Figure 2 and Supplementary Table S2). Currently, all datasets have been generated by the Sanger Institute and consequently there is a direct relationship between the cell model described and the datasets provided, mitigating the risk of mis-identification and genetic drift. Genomic datasets for each model are provided as raw data, typically a link to the appropriate repository, and as processed files or data tables that can be directly downloaded from the website or queried using the available API. The processed files and a description of cancer driver gene status for each model facilitates access for non-informaticians. Furthermore, cell model drug sensitivity data have been provided where available, and the Passport provides links to the associated GDSC webpage for a given model.

Each processed genomic dataset is stored in a dedicated database table. Tables hold the data in long form, with each row a gene along with its associated values. In the case of mutations, there can be multiple rows for any given gene due to the presence of multiple mutations in a gene of a given model. The processed datasets provide gene-level information, rather than segment or transcript, allowing direct integration and cross-comparisons with other datasets within the Passports. All genes have been assigned an internal ID, allowing mapping to current and previous HGNC gene symbols, Ensembl Gene IDs (v91) and other external gene identifiers. All genes with an HGNC-approved symbol as of April 2018 are currently included in the Passports, including those without a protein product. Any dataset values that were mapped to genes without an official gene symbol have been discarded from processing, but continue to be available in raw data downloads.

## DATABASE CONSTRUCTION

### Curation

Model annotation was compiled from information provided by commercial cell banks (ATCC, DSMZ, JCRB and ECACC) academic collaborators, databases (Cellosaurus (19) and CCLE (6)) along with publications (15, 21–33) (Supplementary Table S3). A constrained vocabulary was developed to consolidate model descriptors. The patient, sample, model hierarchy was established by combining information gained during model annotation along with resources cataloging mis-identified and synonymous models (33, 34). In addition STR/fingerprinting profiles gathered internally and during curation were analysed to confirm model-patient associations (33, 34). By default, models sharing common ancestry are assigned to separate samples of a single patient, unless evidence is available indicating otherwise. This relatively conservative approach ensures data can be accurately attributed to the specific cell model, thereby improving reproducibility between users of the same or related models.

### Technologies

The Cell Model Passports resource was constructed using a PostgreSQL (v9.6) database and is hosted at the Wellcome Sanger Institute. The REST API is developed in Python 3.6 using the Flask framework (v0.12) and SQLAlchemy (v1.2). The web interface utilizes the React framework (v16.4), with Bootstrap (v4.0) providing user interface elements and responsive adaptation to screen size.

## ACCESSING AND SEARCHING FOR MODEL INFORMATION AND DATASETS

### Web-based access

The website enables users to search and/or filter for cell models via a variety of features as well as providing access to datasets. The search and filter functions allow users to not only conduct direct queries for an individual model or cancer, but also combine filters to identify a cohort of cell models with a particular set of characteristics. For example, one can filter for non-small-cell lung cancer cell models derived from a metastatic lesion with a mutation in *KRAS* and subsequently access the expression and drug sensitivity data sets for this cohort of models. It is also possible to filter models based on the availability of specific datasets, for example, the subset of breast cancer models with both mutation and gene expression data.

**Figure 3.**
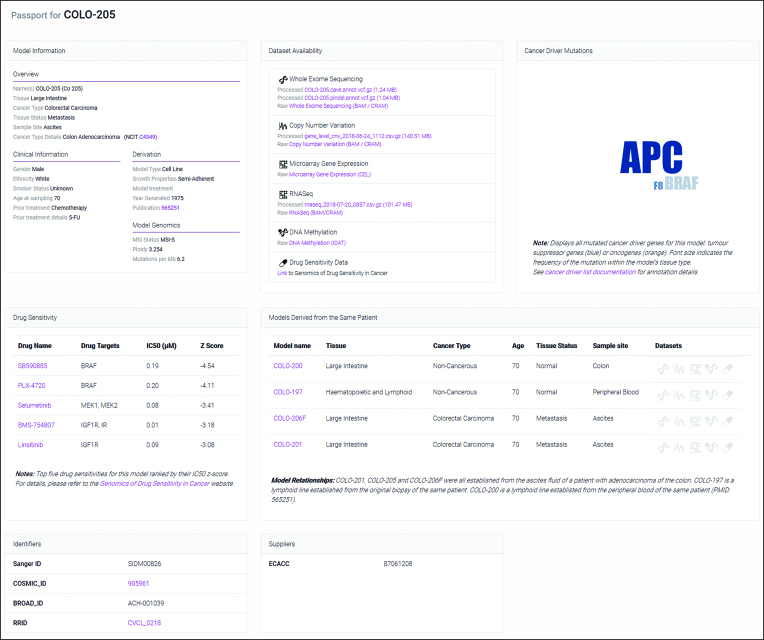
An example of a Passport Model Page. The model features and datasets are provided by the multiple information panels. Links are provided to NCI-Thesaurus definitions as well as raw and processed genomic and drug sensitivity data.

Multiple information panels are displayed on the Model Page (Figure 3). ‘Model Information’ is provided for all models while additional panels are dependent upon availability of relevant information. Cancer driver mutation status for a model is provided as a word cloud, where text size represents the prevalence of mutations in a gene for all cell models of the same tissue type, and the colour differentiates between tumour suppressors and oncogenes. Drug sensitivity data obtained from the GDSC project are also reported showing the compounds to which a model is most sensitive. Dataset highlights such as these assist users to make informed decisions regarding cell models and facilitates further data integration and analysis. Full documentation can be found in the website user guide available on the website.

### API

Programmatic access to the Cell Model Passports database is available through a flexible API. The API can be accessed through HTTPS GET requests to https://api.cellmodelpassports.sanger.ac.uk on specific endpoints. Requests should follow the JSONAPI v1.0 standard (jsonapi.org), though a few additions are available to accommodate search and aggregations. Responses strictly follow the JSONAPI v1.0 standards. Endpoints are available for lists of patients, samples, models, identifiers, files, all processed datasets and cancer driver mutations. Requests can have modifiers that allow users to select, filter and sort the results and control pagination. To facilitate access to available resources, all relationships between resources are fully annotated within the API, including API URLs to related resources. Full documentation and examples can be found in the API user guide available on the Passports website.

### Data files

Data files are hosted on a robust S3 object store platform or in third party repositories; the Cell Model Passport database contains links to these files together with associated metadata. There are two categories of files, each with their own database table: generic files and model files. Model files are tied to a specific model and are currently used for model-specific datasets, images, documentation or authentication details. Generic files hold data for multiple models or are files not otherwise associated with an individual model.

## DISCUSSION

The Cell Model Passport is a centralized cell model hub, providing a platform for researchers to investigate key patient and sample information, manually and programmatically access multiple genetic and functional datasets, and facilitate integration of data from different sources through mapping of unique identifiers.

Given the widespread usage of cancer cell models several resources exist that hold cell model information such as Cellosaurus (19), PharmacoDB (35), cBioPortal (36) and COSMIC (20). Nonetheless, the Cell Model Passports integrate and build upon these to provide new functionality. First, Cellosaurus and cBioPortal do not provide cell model functional information, while PharmacoDB does not contain genomic annotation of the cell models and has limited sample/model information. Second, cBioPortal and COSMIC are primarily focused on patient tumour genomic data. Third, neither cBioPortal, PharmacoDB nor COSMIC curate samples to map unique identifiers or link samples and models from the same patient, which can be important for identifying mis-identified or cross-contaminated samples. To the best of our knowledge, there are no other web-resources providing a centralized portal with cell model relationship information, access to associated datasets and annotation of cancer gene status and drug sensitivity. Thus, the Cell Model Passport database builds upon and extends the functionality of existing resources.

In addition to the existing deep characterization, cell lines are continually being used in new initiatives such as the Dependency Map at Sanger (depmap.sanger.ac.uk), generating additional datasets, such as whole-genome CRISPR-knockout screens, calling of fusion transcripts and additional drug response data. The Cell Model Passports will incorporate these newly generated datasets to facilitate their utilization for scientific research. The Passports currently focus on high quality and consistent datasets generated by the Wellcome Sanger Institute, but in the future will expand to include cell models and datasets generated by external resources while maintaining an emphasis on quality and curation. Furthermore, with recent technological advancements (e.g. organoid models and conditionally reprogrammed cell lines), a new generation of cancer cell models is being derived to allow full representation of the histopathological and molecular diversity of tumours (37–42). The Sanger Institute is a member of the Human Cancer Model Initiative, which aims to make a resource of highly annotated cancer models, and the Cell Model Passports will ensure that model annotation, relationships and open access to experimental data is preserved from the outset.

In summary, the Cell Model Passports provide a resource to reduce the mis-identification of cell models, facilitate data integration and subsequently improve experimental reproducibility in cancer research and related fields. We anticipate that the Passports will be widely used to inform the selection of cancer models and to provide user-friendly access to cell model relationship information as well as clinical, genomic and functional datasets.

## Supplementary Material

Supplementary Data
